# Tomato and its relatives are breaking the genomics barriers

**DOI:** 10.1093/jxb/eraf298

**Published:** 2025-07-03

**Authors:** Laura Ellen Rose, Zahra Zangishei, Alisdair R Fernie, Björn Usadel

**Affiliations:** Faculty of Mathematics and Natural Sciences, Institute of Population Genetics, CEPLAS, Heinrich Heine University Düsseldorf, 40225 Düsseldorf, Germany; Faculty of Mathematics and Natural Sciences, Institute of Biological Data Science, CEPLAS, Heinrich Heine University Düsseldorf, 40225 Düsseldorf, Germany; Max Planck Institute of Molecular Plant Physiology, 14476 Potsdam-Golm, Germany; Faculty of Mathematics and Natural Sciences, Institute of Biological Data Science, CEPLAS, Heinrich Heine University Düsseldorf, 40225 Düsseldorf, Germany; Institute of Bio- and Geosciences, IBG-4, Bioinformatics, BioSC, CEPLAS, Forschungszentrum Jülich, 52428 Jülich, Germany; University of California, Davis, USA

**Keywords:** Exotic germplasm, genomics, metabolomics, population genetics, quantitative trait loci, tomato, wild relative

## Abstract

Recent advances in high-quality genome sequencing have revolutionized research in the tomato clade (*Solanum* section *Lycopersicon*), enabling the generation of long-read and even chromosome-scale assemblies for cultivated tomato and its wild relatives. These data have shed light on tomato domestication and population genetics and have facilitated breeding using exotic germplasm. This review summarizes progress in tomato genomics, focusing on the diversity of section *Lycopersicon* and its function as a reservoir of stress-tolerance genes, including drought tolerance from *Solanum pennellii* and pathogen resistance from *Solanum habrochaites* and *Solanum chilense*. We catalog important genetic resources, including introgression lines and multi-parent advanced generation inter-cross (MAGIC) populations, which have allowed the dissection of important traits via the mapping of quantitative trait loci, including those involved in primary and secondary metabolism. We also explore the metabolic diversity of wild and domesticated tomato species and discuss how this has led to gene identification. Finally, we show that tomato genomics will continue to accelerate, given the increasing availability and accessibility of genomics technology, exotic germplasm, and mapping populations, which can be leveraged using advanced genome-editing approaches.

## Teaser

Recent breakthroughs in genome sequencing have transformed tomato research, unlocking key wild germplasm traits. This review outlines how these well-characterized genetic resources can be used to improve tomato research.

## Introduction

In the past decade, genomics has shone a spotlight on wild tomato species and their use as donors of exotic germplasm. This was driven by the explosion of innovation in next-generation sequencing. Second-generation sequencing, which refers to short-read sequencing applied to clonal DNA amplicons, was the main driver behind the published genomes of cultivated tomato (*Solanum lycopersicum*), its close relative *Solanum pimpinellifolium* (Tomato Genome Consortium, 2012), and its more distant cousin *Solanum pennellii* (Bolger *et al*., 2014). Additional milestones include transcriptomic analysis in several wild tomato species showing footprints of selection (Koenig *et al.*, 2013), and the analysis of genetic diversity within the tomato clade (100 Tomato Genome Sequencing Consortium *et al.*, 2014), which revealed much lower variation in domesticated tomato compared with wild relatives. More recently, long-read sequencing facilitated the construction of the first nanopore assembly of a plant genome, namely *S. pennellii* (Schmidt *et al*., 2017). This was followed by the release of the first tomato pangenome, combining information from several hundred tomato genomes (Gao *et al.*, 2019), nanopore-based analysis of structural variation across multiple tomato species and relatives (Alonge *et al.*, 2020), and a graphical tomato pangenome that helped to explain heritability (Zhou *et al.*, 2022). These efforts have culminated in the assembly of a tomato super-pangenome that also incorporates wild species (Li *et al.*, 2023). These endeavors required large consortia for *de novo* genome assembly, annotation, and analysis.

Third-generation sequencing, which refers to long-read sequencing involving single DNA molecules, is the state of the art for *de novo* genome analysis (Jiao and Schneeberger, 2017). This is because the refinement of such techniques significantly reduces costs and complexity, bringing genome sequencing within the capability of even small laboratories (Dumschott *et al.*, 2020; Pucker *et al.*, 2022). Early iterations of the two prevalent long-read technologies—nanopore sequencing (Oxford Nanopore Technologies; ONT) and single-molecule real-time sequencing, commercialized as PacBio sequencing by Pacific Biosciences—were hindered by high error rates of ∼10% (Watson and Warr, 2019). However, technological improvements and machine learning algorithms for data analysis have reduced error rates to ∼1% (i.e. Q20) and below. ONT increases accuracy not only by applying machine learning but also by identifying consecutively sequenced reads stemming from both strands of a double-stranded molecule (duplex reads). This has reduced the error rate for these read pairs to ∼0.1% (i.e. Q30). However, these events represent only ≤20% of all sequenced read pairs. In contrast, PacBio uses a special library preparation technique to sequence the same circular molecule many times, thus consistently reducing the typical error rate to ∼0.1%. One drawback of this approach is that PacBio reads are typically 15–20 kb in length, whereas nanopore reads can be much longer, up to dozens of kilobases when properly selected, resulting in so-called ultra-long fragments (Lu *et al.*, 2024).

Owing to competition between the nanopore and PacBio technologies, they offer similar outputs: up to 90 Gb for PacBio sequencing and 50–120 Gb for nanopore sequencing, sometimes even more. Nanopore sequencing is more economical in terms of flow cells and library preparation (approximately €1000 per run, with additional discounts for higher order volumes, and approximately €20 000 upfront investment for high output sequencing). PacBio sequencing is not much more expensive per run, but the upfront investment is significantly higher at several hundred thousand euros. PacBio has released a new VEGA sequencer that costs less than €200 000 to attract smaller laboratories, but the throughput is relatively lower (60 Gb per run). Given the typical size of tomato genomes, both technologies produce good draft genomes from a single flow cell.

In addition to *de novo* genome assembly, both long-read technologies improve the analysis and identification of structural variants in comparison to short-read sequencing (Alonge *et al.*, 2020). Both can also call modified bases at no extra cost, mainly 5-methylcytosine in the CG context for PacBio and in all contexts for nanopore sequencing. This allows the direct analysis of epigenetic modifications, that is, those that may influence tomato fruit ripening (Zhong *et al.*, 2013) and flavor loss induced by chilling (Zhang *et al.*, 2016), and could also be associated with tomato domestication (Guo *et al.*, 2023). More recent technological advances allow the molecular footprinting of chromatin architecture relying on 6-methyladenine modification and identification (Stergachis *et al.*, 2020; Leduque *et al.*, 2024), although in the case of PacBio sequencing the identification of this modification is restricted to their larger instruments and is not yet available on the VEGA platform. As a sign of continuing innovation, PacBio recently announced a new sequencing chemistry that improves accuracy, and ONT has enhanced its machine learning workflow. Both technologies can also generate Hi-C-like chromosome proximity information by leveraging multi-contacts (Koren *et al.*, 2024) to construct chromosome-scale assemblies.

Third-generation sequencing technologies have evolved hand in hand with bioinformatics algorithms for sequence analysis and assembly, such as the popular assemblers hifiasm (Cheng *et al.*, 2021) and Verkko (Rautiainen *et al.*, 2023), as well as assemblers specifically developed for nanopore data such as FLYE (Kolmogorov *et al.*, 2019) and nextdenovo (Hu *et al.*, 2024). The complementarity of the two long-read technologies, one providing more accurate reads and the other generating longer reads, has facilitated the analysis of a tobacco mosaic virus resistance locus from *Solanum peruvianum* introgressed into domesticated tomato (van Rengs *et al.*, 2022). Accordingly, most workflows now rely on hifiasm and/or Verkko so that they can profit from both technologies at once. Further developments allow not only the use of more precise nanopore duplex reads, but also the self-correction of nanopore data (Stanojevic *et al.*, 2024). This enables telomere-to-telomere assemblies based on nanopore data alone. Such assemblies may be possible for tomato with sequencing alone, depending on the genome architecture and heterozygosity of the selected species or accession (Koren *et al.*, 2024). Furthermore, tools that integrate artificial intelligence (AI) for genome annotation (Holst *et al.*, 2023) also allow the fully automated structural annotation of tomato genes or to classify transposable elements (Yan *et al.*, 2020). Finally, nanopore ultra-long reads and/or adaptive sampling offer promising solutions for gap filling and sequence resolution in complex plant genomes, as demonstrated by many near-complete telomere-to-telomere publications (Lu *et al.*, 2024). While ONT long-read data require the extraction of high-quality DNA, which can be challenging, multiple extraction methods have been developed for solanaceous species (Alonge *et al.*, 2020; Vilanova *et al.*, 2020), and commercial kits from Macherey-Nagel were shown to provide DNA of sufficient quality (Chalupowicz *et al.*, 2019).

Hence, it is now possible to obtain very good genomic representations of tomato and its wild relatives by relying on sequencing progress. At the same time, bioinformatics and AI-based tools will allow progressively better genome assemblies. For nanopore data, improvements have been achieved by making use of AI-based data correction (Stanojevic *et al.*, 2024). Hence, the use of AI can now be focused on the hitherto difficult gene-finding process (Holst *et al.*, 2023) and improved algorithms for defining orthologs, for example, in orthofinder (Emms and Kelly, 2019) can be employed. In addition, the development of graphical pangenomes and super-pangenomes can help in interpreting and analyzing populations.

## Tomato species and their close relatives in the genomics area

Section *Lycopersicon* comprises 13 species, according to some of the most recent taxonomic studies (Peralta *et al.*, 2005; Rodriguez *et al.*, 2009; Pease *et al.*, 2016). Most are diploid and feature 12 highly syntenic chromosomes (2*n* = 2*x* = 24) with only minor cytological differences (Anderson *et al.*, 2010) and a genome size of 900–1200 Mb (Arumuganathan and Earle, 1991; Li *et al.*, 2023) (Table 1). The section has four major groups: *Lycopersicon* (domesticated tomato and closely-related red-fruited tomatoes), *Arcanum* (containing both selfing and outcrossing green-fruited tomatoes), and the two groups *Peruvianum* and *Hirsutum*, which will be discussed later as exotic germplasm donors. Metabolomics analysis has been applied to many of these species (Schauer *et al.*, 2005; Schwahn *et al.*, 2014; Tohge *et al.*, 2020), allowing a high coverage of primary and secondary metabolites. Significant quantitative variation was observed for some primary metabolites, whereas others, such as alanine, glycine, 2-oxoglutarate and cell-wall sugars, were somewhat less variable (Schauer *et al.*, 2005). Both qualitative and quantitative variation has been observed for secondary metabolites, with some differences being species dependent (Tohge *et al.*, 2020).

**Table 1.** Genomic resources available to study tomato genomics

Species	Accession	Approach	Genome size (Mbp)	Status	Anchoring rate (%)	Repeats (%)	Predicted genes	Citation
*Solanum lycopersicum*	Heinz 1706 (SL4.0)	PacBio, Illumina, BioNano, Hi-C	782.52	Chromosome scale	98.80	64.19	34 075	Hosmani *et al.*, 2019 (Preprint)
	Heinz 1706	PacBio, Illumina, BioNano, Hi-C	801.8	Chromosome scale	99.8	61.27	36 648	Zhou *et al*., 2022
*Solanum pimpinellifolium*	LA1589	Illumina	739	Contig level (309 180)				Tomato Genome Consortium, 2012
	LA0480	Illumina	811.30	Scaffold level (163 297)		59.50	25 970	Razali *et al.*, 2018
	LA2093	PacBio, Hi-C	807.60	Chromosome scale	99.00	67.30	35 761	Wang *et al.*, 2020
	LA1547	PacBio, Hi-C	803.0	Chromosome scale	94.78	72.77	33 427	Li *et al.*, 2023
	LA1589	PacBio, Optical Mapping	833	Chromosome scale	98.8	74.47	41 449	Han *et al.*, 2024
*Solanum galapagense*	LA0317	PacBio, Hi-C	859.90	Chromosome scale	94.36	73.15	33 108	Yu *et al.*, 2022
	LA0436	PacBio, BioNano, Hi-C	805.00	Chromosome scale	99.56	71.45	32 773	Li *et al.*, 2023
*Solanum cheesmaniae*	LA1406, LA1407, LA1409, LA0528B	nanopore		Advanced long read				Alonge *et al*., 2020
*Solanum chmielewskii*	LA1028	PacBio and Hi-C	770.01	Chromosome scale	95.44	72.26	31 613	Li *et al.*, 2023
*Solanum arcanum*	LA2157	Nanopore, Illumina, Hi-C	855.68	Chromosome scale	99.20	48.73	33 489	Jiang *et al.*, 2023
*Solanum neorickii*	LA0247	PacBio, Hi-C	778.27	Chromosome scale	94.07	72.74	32 831	Li *et al.*, 2023
*Solanum peruvianum*	LA0446	PacBio, Hi-C	867.47	Chromosome scale	91.90	73.83	31 877	Li *et al.*, 2023
*Solanum corneliomulleri*	LA1331	PacBio, Hi-C	876.90	Chromosome scale	88.60	74.49	31 692	Li *et al.*, 2023
*Solanum chilense*	LA3111	Illumina	913.89	Contig level (81 307)			25 885	Stam *et al.*, 2019
	LA1969	PacBio and Hi-C	916.71	Chromosome scale	88.11	73.70	34 375	Li *et al.*, 2023
	LA1972	PacBio, Illumina, BioNano, Hi-C, Chromium	902.00	Chromosome scale	96.00	62.63	32 972	Molitor *et al.*, 2024
*Solanum habrochaites*	LA0407	PacBio, Hi-C	950.70	Chromosome scale	95.42	74.18	33 567	Yu *et al.*, 2022
	LA1777	PacBio and Hi-C	959.70	Chromosome scale	86.07	69.05	32 386	Li *et al.*, 2023
*Solanum pennellii*	LA0716	Illumina	942.60	Chromosome scale	97.10	61.16	44 965	Bolger *et al*, 2014
	LA5240	ONT, Illumina	915.60	Long draft	97.10	68.40		Schmidt *et al*, 2017
*Solanum lycopersicoides*	LA2951	PacBio, Hi-C	1200.8	Chromosome scale	89.50	68.00	37 938	Powell *et al*, 2022
	LA2951	PacBio, Hi-C	1200.0	Chromosome scale	92.23	71.81	32 295	Li *et al.*, 2023
*Solanum sitiens*	LA1974	Illumina, PacBio	1245.0	Scaffold level (1483)			31 164	Molitor *et al*., 2021
*Solanum juglandifolium*	N/A	N/A	N/A	N/A	N/A	N/A	N/A	N/A
*Solanum ochranthum*	PI23051901	PacBio and Hi-C	956.17	Chromosome scale		64.98	42 164	Wu *et al.*, 2023

In addition, the reader is referred to solgenomics.net and the large-scale study of Alonge *et al*. (2020_ for long read data.

Interestingly, many tomato species and accessions can be crossed with cultivated tomato, indicating weak interspecific reproductive barriers that allow the introgression of exotic material with desirable traits (Tanksley and McCouch, 1997; Zamir, 2001). The trailblazing work of C.M. Rick beginning in the 1960s (Rick, 1963) set the stage for studies on the source and strength of interspecific barriers within the tomato clade. The fact that many wild tomato species can readily be distinguished at the morphological and genetic levels implies that interspecific barriers for many species pairs are intact. However, the source of these barriers differs depending on the time of speciation and the mating system of the focal species. Furthermore, interspecific barriers are often asymmetrical. Selfing species, such as *S. lycopersicum*, can often serve as the maternal plant, accepting pollen from heterospecific pollen donors (whether the pollen stems from selfing or self-incompatible species). In contrast, pollen from selfing species typically fails to fertilize the ovules of self-incompatible species. Therefore, the receptiveness of stigmas to heterospecific pollen differs for selfing and self-incompatible species, typically summarized as the ‘SI × SC rule’ (Baek *et al.*, 2015). Within tomato species that are polymorphic for mating systems, self-compatible accessions are usually located on the periphery of the species range, where conspecific pollen and pollinators may be limited in availability. Accordingly, although the stigmas of selfing species would theoretically be receptive to heterospecific pollen, morphological changes (e.g. stigma shortening, which is also linked to seed-setting efficiency when pollen is limiting) may also form a reproductive barrier, reducing the rate of introgression between self-compatible and self-incompatible species. These intraspecific and interspecific barriers have recently been reviewed (Moreels *et al.*, 2023).

### Red-fruited tomatoes, the *Lycopersicon* group

The *Lycopersicon* group of red-fruited tomatoes includes the cultivated tomato, domesticated lineages of *S. lycopersicum* and *S. pimpinellifolium*, as well as the species *Solanum cheesmaniae* and *Solanum galapagense*, both of which are endemic to the Galapagos Islands. The ranges of *S. lycopersicum* and S*. pimpinellifolium* are sympatric in northern Peru and extend to Ecuador, whereas those of *S. galapagense* and *S. cheesmaniae* are restricted to the Galapagos Islands. Among these species, the outcrossing rate appears highest for *S. pimpinellifolium* and lowest for *S. cheesmaniae.* The frequency of outcrossing correlates with overall intraspecific genetic diversity (Beddows *et al.*, 2017), with the highest intraspecific variation observed in *S. pimpinellifolium* and the lowest in *S. cheesmaniae*.

Genomic data, including large-scale population genomics data, has helped to shed light on tomato domestication. Domestication was probably a two-stage process, with an initial round of selection in South America followed by a second round in Mesoamerica (Blanca *et al.*, 2012, 2015). This explains why the modern tomato *S. lycopersicum* is split into varieties *lycopersicum* (SLL) and *cerasiforme* (SLC) (Blanca *et al.*, 2015). The two-step domestication hypothesis was recently supported by the analysis of the tomato pangenome (Gao *et al.*, 2019). This revealed the loss of ∼200 genes in *S. pimpinellifolium* from northern Ecuador, which is believed to be basal to *S. lycopersicum*, and additional gene loss continuing through the subsequent domestication of SLC on its way to Mesoamerica (Gao *et al.*, 2019). SLC may have split from *S. pimpinellifolium* as recently as 78 000 years ago in Ecuador, probably predating the presence of humans in the Americas, whereas the domestication of modern SLL took place in Mexico less than 10 000 years ago (Razifard *et al.*, 2020). Haplotype analysis (Blanca *et al.*, 2022) demonstrated that Peruvian and Ecuadorian SLC is an admixture of Mesoamerican SLC and *S. pimpinellifolium*, and that domesticated SLC migrated north to Mexico and became SLL. These data, highlighting the origin of modern tomato, were obtained by analyzing large populations of red-fruited tomato using population genomics approaches. With the availability of more and more genomes of other tomato species (see below) and by analyzing larger populations, the more distant history of tomato might become clearer as well. This could be achieved by tracking gene losses and gains as well as evolutionary bottlenecks using population genomics approaches.

Analysis of the *de novo* genome assembly of the wild accession *S. pimpinellifolium* LA0480 provided evidence for the enrichment of genes involved in biotic and abiotic stress tolerance, including a higher copy number of genes involved in inositol metabolism, which is potentially responsible for its higher salt tolerance (Razali *et al.*, 2018). Given the domestication history discussed above, and current breeding targets, it is unsurprising that signals of introgression from *S. pimpinellifolium* are detected in domesticated tomato (Alonge *et al.*, 2020; Wang *et al.*, 2020). Furthermore, analysis of the red-fruited tomatoes yielded several important insights into fruit quality traits. To name just some examples, in the population used for the pangenome analysis (Zhou *et al.*, 2022) a *TREHALOSE-PHOSPHATE PHOSPHATASE* was identified as important for sugar accumulation. This was also found through system biology approaches (Li *et al.*, 2021). In addition, a deletion in the promoter of the sugar transporter *STP1* (Wang *et al.*, 2023) changed fruit soluble sugar content. Yet another insertion/deletion (InDel) in *TOMATO FRUIT MALATE 6* (i.e. *Al-ACTIVATED MALATE TRANSPORTER9*) influenced the organic acid malate (Ye *et al.*, 2017), whereas an InDel in the promoter region of a *bHLH* gene influenced ascorbate content (Ye *et al.*, 2019).

Three multi-parental advanced generation intercross (MAGIC) populations have been established between tomato and some of its closest wild relatives, the first comprising lines of SLL and SLC (Pascual *et al.*, 2015). A further MAGIC population included seven cultivated accessions and *S. cheesmaniae*, in which the founders were selected based on their stress tolerance, yield, and resilience (Campanelli *et al.*, 2019). The most recently established MAGIC population comprises four SLC lines and four *S. pimpinellifolium* founders selected for diversity and stress tolerance (Arrones *et al.*, 2024) and for which all founder genomes have been sequenced (Gramazio *et al.*, 2020). A complete genomic library of introgression lines (ILs) has also been developed from a cross between *S. pimpinellifolium* TO-937 and *S. lycopersicum* cv. Moneymaker (Barrantes *et al.*, 2014) (Table 2). In addition, a large recombinant inbred line (RIL) population has been developed from a cross between *S. pimpinellifolium* LA2093 and *S. lycopersicum* cv. NCEBR-1 (Ashrafi *et al.*, 2009) and used to localize, for instance, a lycopene QTL (Ashrafi *et al.*, 2012) (Table 2). While the MAGIC populations offer the advantage of introducing multiple wild allele donors that can be explored all at once, the IL and RIL populations can help focus on individual traits found in the one wild donor parent.

**Table 2.** Some major populations available to the community

Exotic donor	Type	Lines	Citation
*Solanum pimpinellifolium, Solanum lycopersicum* var. *cerasiforme*	MAGIC	354 lines	Arrones *et al.*, 2024
*S. pimpinellifolium* TO-937	Introgression lines	53 lines	Barrantes *et al*., 2014
*S. pimpinellifolium* LA2093	Recombinant inbred lines	170 F_7_ lines	Ashrafi *et al*., 2009
*Solanum neorickii* LA2133	Introgression lines	107 lines	Brog *et al.*, 2019
*Solanum pennellii* LA0716	Introgression lines and backcross inbred lines	76 introgression and 446 BIL lines	Ofner *et al*., 2016
*S. pennellii* LA5240	Backcross inbred lines	1400 total	Torgeman *et al.*, 2024
*Solanum habrochaites* LA1777	Near isogenic lines and backcross recombinant inbred lines	99 lines	Monforte and Tanksley, 2000
*S. habrochaites* LYC4	Introgression lines	30 lines	Finkers *et al.*, 2007
*Solanum sitiens* LA4331 and LA1974	Introgression lines	56 lines	Chetelat *et al*., 2019
*Solanum lycopersicoides* LA2951	Introgression lines	56 primary and 34 additional lines	Canady *et al.*, 2005, 2006

The close relatives of domesticated tomato have been subjected to deeper analysis (Strickler *et al.*, 2015). A subsequent study used single-nucleotide polymorphisms (SNPs) as markers to analyze the genetic diversity of *S. galapagense* and *S. cheesmaniae*, revealing that *S. pimpinellifolium* accessions cluster with those of *S. lycopersicum*, while accessions of *S. galapagense* and *S. cheesmaniae* are clearly distinguished at the genetic level (Pailles *et al.*, 2017). Additionally, the study revealed that there is relatively little genetic diversity in *S. galapagense*, in line with earlier studies (Koenig *et al.*, 2013). However, *S. cheesmaniae* showed greater genetic diversity and clear differences between accessions collected in eastern and western Galapagos islands. However, few *S. pimpinellifolium* accessions were analyzed because the main focus of the study was the population structure of the endemic Galapagos species. Pangenome analysis based on presence/absence gene variation was recently applied to the *Lycopersicon* group, but only three and five accessions of *S. cheesmaniae* and *S. galapagense*, respectively, were included (Gao *et al.*, 2019). Nevertheless, the study revealed a potential trend for gene loss during domestication as well as a stronger influence on gene promoters during domestication than during the subsequent improvement. In a more recent study, loci probably responsible for the purple fruit pigmentation of *S. galapagense* accession LA1141 were studied in a targeted IL population (Fenstemaker *et al.*, 2022), and the large-scale analysis of fruit quality traits has provided insight into fruit organoleptic qualities (Tieman *et al.*, 2017; Zhang *et al.*, 2024).

**Fig. 1.**
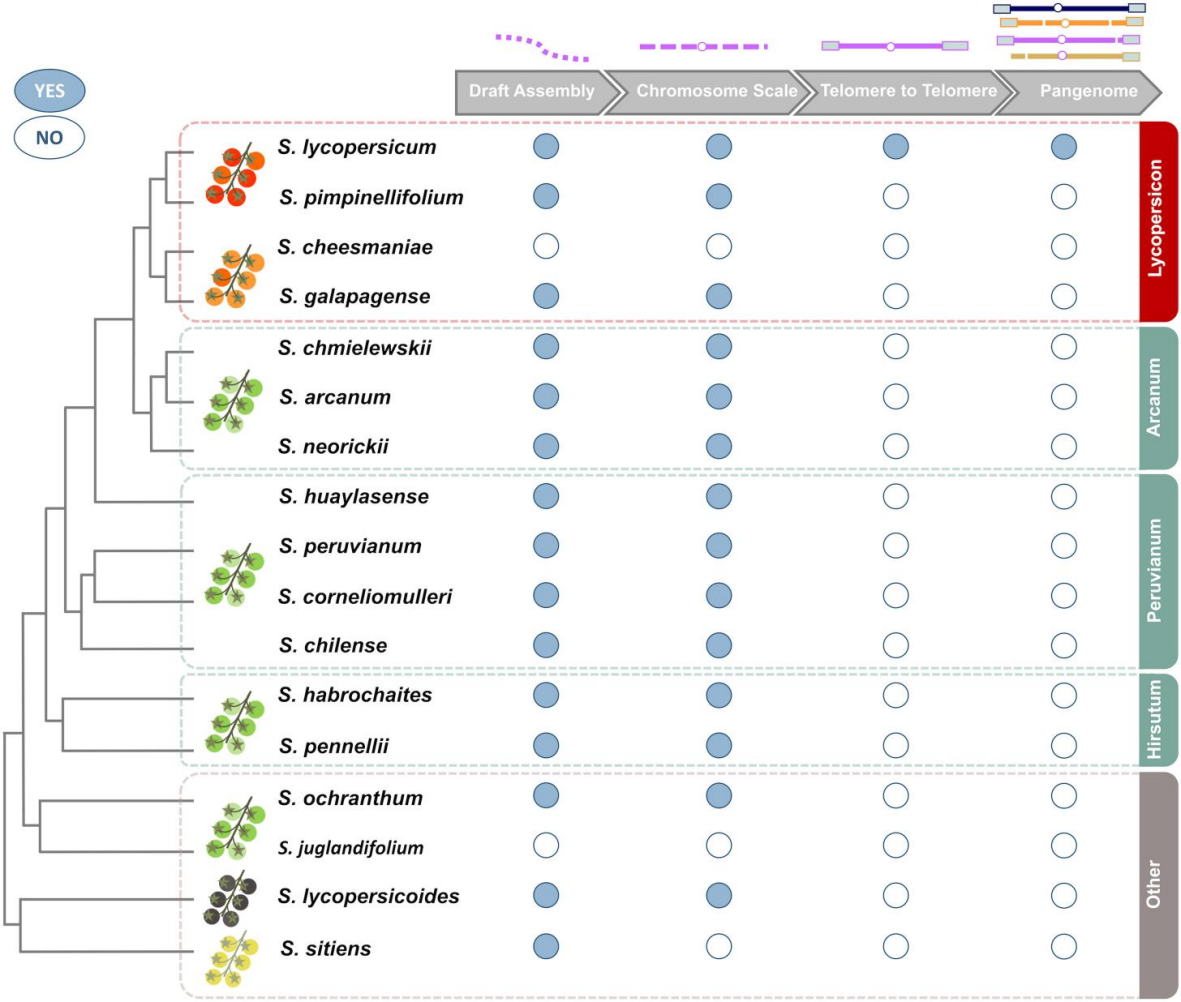
Current stage of tomato and wild relative genome sequencing.

Chromosome-scale genomes based on PacBio and Hi-C technologies are now available for all tomato species except *S. cheesmaniae* (Wang *et al.*, 2020; Yu *et al.*, 2022; Li *et al.*, 2023), and long-read structural variation based on nanopore data is available for *S. cheesmaniae* (Alonge *et al.*, 2020) (Table 1; Fig. 1).

### The *Arcanum* group

The *Arcanum* group comprises three species, *Solanum arcanum*, *Solanum chmielewskii*, and *Solanum neorickii*, and shows a range of mating systems. These species are green-fruited but share a more recent common ancestor with the red-fruited clade than with other green-fruited species (Bedinger *et al.*, 2011; Pease *et al.*, 2016). The range of *S. chmielewskii* is restricted to southern Peru, whereas *S. neorickii* extends from southern Peru to Ecuador. Although morphologically very similar, these sister species are distinguished by flower size, fruit size, and rate of outcrossing: *S. chmielewskii* has larger flowers, larger fruits, and more outcrossing behavior. As early as 1940, a variety of what was then thought to be *S. peruvianum* was found and named humifusum (Muller *et al.*, 1940). These individuals largely corresponded to the species now recognized as *S. arcanum*, which is distinguished from others in the clade by its unbranched inflorescences, straight anther tubes, and short styles (Muller *et al.*, 1940; Rick, 1986). Test crosses between *S. peruvianum* and the humifusum variety (*S. arcanum*) showed reduced seed set, suggesting that some breeding barriers existed between these individuals.

Rick (1986) recognized four more or less reproductively isolated groups based on reciprocal test crosses: Chotano-humifusum, Chamaya-Cuvita, Marañón, and typical *S. peruvianum*. What is now recognized as *S. arcanum* corresponds to the individuals from the first three groups (Peralta *et al.*, 2008). The placement of *S. arcanum* within the tomato clade was initially difficult as there were only one or two accessions from which to draw conclusions (100 Tomato Genome Sequencing Consortium *et al.*, 2014; Pease *et al.*, 2016). However, more recent population genomics analyses (Beddows *et al.*, 2017) placed it close *to S. neorickii*. More recently, crossing and transcriptomics analysis revealed that the ancestors of the self-incompatible Marañón group may have given rise to both *S. chmielewskii* and *S. neorickii* (Florez-Rueda *et al*., 2021). The authors reported a distinction between northern and southern Marañón accessions, the southern ones being associated with *S. chmielewskii* and the northern ones with *S. neorickii* (Florez-Rueda *et al.*, 2021). The *S. arcanum* LA2157 genome has also been used to identify a heat-stable root knot nematode resistance gene (Jiang *et al.*, 2023).

Both *S. chmielewskii* and *S. neorickii* were characterized in the metabolomic studies described above. *S. chmielewskii* was found to contain high levels of starch and sucrose (Schauer *et al.*, 2005), as well as qualitative and quantitative differences in the levels of steroidal glycoalkaloids (SGAs) and phenylpropanoids (Schwahn *et al.*, 2014; Tohge *et al.*, 2020). However, the genomic basis of these differences has been explored only in a sink–source interaction study on a set of *S. chmielewskii* ILs, revealing considerable differences in amino acid transport (Do *et al.*, 2010). Analysis of *S. chmielewskii* populations eventually led to the isolation of an acid invertase gene linked to sucrose accumulation (Klann *et al.*, 1993). Compared with cultivated tomato, *S. neorickii* produced similar levels of most primary metabolites, but higher levels of serine in the leaves (Schauer *et al.*, 2005). In contrast, *S. neorickii* and cultivated tomato showed many differences in secondary metabolism, with distinct, species-dependent profiles of neorickiiside-SGAs (Schwahn *et al.*, 2014) and. to a lesser extent. phenylpropanoids (Tohge *et al.*, 2020).

A recently created backcrossed introgression line (BIL) population of *S. neorickii* comprised 107 lines derived of the progeny of the wild species *S. neorickii* (LA2133) and the cultivated tomato (TA209) (Brog *et al.*, 2019). These lines feature an average of 4.3 introgressions, facilitating the partitioning of the genome into 340 bins to accelerate trait mapping. This was demonstrated by the recent mapping of genes encoding phenylalanine ammonia-lyase and cystathionine gamma lyase, followed by validation in an F_2_ population and overexpression lines (Brog *et al.*, 2019) (Table 2). Further studies are needed to dissect the secondary metabolites, volatiles, and lipids in this population as well as other beneficial traits, but the large number of bins would allow a relatively good resolution.

### The diverse *Peruvianum* group

*Solanum peruvianum*, the most diverse species in the tomato clade, seems to feature two groups (Rick, 1963; Nakazato *et al.*, 2012). This substructure was recently dissected by sequencing individual transcriptomes from 18 populations across the species range (Beddows *et al.*, 2017). One group comprises individuals from low-elevation populations along the coast and/or lomas formations of southern Peru, whereas the other comprises individuals from non-coastal central Peruvian populations. The coastal group was previously reported to display less variation in shape, size, and habit between populations, but greater variation within any given population, whereas non-coastal populations were more restricted (Rick, 1963). Dispersal, and hence gene flow, between populations along the Andean river drainage zones may be strongly limited by geographical barriers, perhaps driving the observed population differentiation. Gene flow may also be restricted by other reproductive barriers, with crosses between plants from these two demes showing low levels of inter-fertility (Beddows *et al.*, 2017).

*Solanum chilense* is self-incompatible and has adaptations to cope with arid habitats (Moyle, 2008). The split between *S. chilense* and its sister species *S. peruvianum* may have occurred as recently as 0.55 million years ago (Mya), and the absence of fixed differences suggested that speciation occurred under residual gene flow (Städler *et al.*, 2008). However, more recent analysis indicates that the split between these species occurred 1.25–2 Mya (Pease *et al.*, 2016; Beddows *et al.*, 2017), which would be explicable if the earlier study included individuals of hybrid origin (Städler *et al.*, 2008). Given the number of species in the tomato clade, their close relationships, and frequent inter-fertility, there may be a number of cryptic hybrid populations. An earlier study based on the analysis of 30 genes from 23 *S. chilense* accessions suggested that accession LA1930 might be a hybrid (Böndel *et al.*, 2015). However, the definitive ancestry of the populations (including LA1930) restricted to the Acari river drainage zone are still unresolved and future long-read genomics approaches will likely solve these.

Populations of *S. chilense* show complex patterns of pathogen resistance (Stam *et al.*, 2017; Kahlon *et al.*, 2020, 2023). Furthermore, signatures of local adaptation possibly associated with drought or cold stress were found in abiotic stress-related genes (Xia *et al.*, 2010; Mboup *et al.*, 2012; Fischer *et al.*, 2013). Some stress-specific adaptations in *S. chilense* are present in tetraploid accessions (Rick, 1990).

Recent taxonomic studies of wild tomato recognize *Solanum corneliomulleri* as a taxon distinct from *S. peruvianum* (Peralta *et al.*, 2005). *S. corneliomulleri* was first described as *Lycopersicon glandulosum* (Muller *et al.*, 1940), but MacArthur and Chiasson (1947) and later Rick (1963) demonstrated the compatibility between *L. glandulosum* and *S. peruvianum*. Therefore, *L. glandulosum* was renamed *L. peruvianum* var. glandulosum and later recognized as a race of *S. peruvianum* (Warnock, 1988). Some studies found somewhat lower evidence of genetic or ecological differentiation between *S. corneliomulleri* and *S. peruvianum* (Rodriguez *et al.*, 2009; Zuriaga *et al.*, 2009; Nakazato *et al.*, 2012; Labate *et al.*, 2014; Pease *et al.*, 2016). This should be addressed by long-read genome sequencing to improve on the more error-prone transcriptomics data. Indeed, a super-pangenome of the tomato clade generated by long-read sequencing placed *S. corneliomulleri* closest to *S. peruvianum*, but all genomes of the *Peruvianum* group (*S. peruvianum*, *S. chilense*, and *S. corneliomulleri*) were relatively heterozygous (Li *et al.*, 2023). Regardless of its status, multiple *S. corneliomulleri* accessions are associated with tomato yellow leaf curl virus resistance (Yan *et al.*, 2018).

A second taxon, named *Solanum huaylasense*, was reported to be morphologically distinct from *S. peruvianum* (Peralta *et al*., 2005). Transcriptomic analysis of six *S. huaylasense* individuals suggested that the species is polyphyletic (Beddows *et al.*, 2017). The distribution of variation in the genomes of these individuals uncovered some degree of admixture (or genetic similarity to other species) in each individual (Beddows *et al.*, 2017). However, the source and degree of the admixture differed for each individual. One group had a genome with nearly equal components of *S. peruvianum* and *S. arcanum*, one had a low amount of admixture with *S. arcanum*, and the final one showed the greatest similarity to individuals of *S. peruvianum*.

### The *Hirsutum* group

The *Hirsutum* group, which is the group most distantly related to domesticated tomato, comprises two species: *S. pennellii and Solanum habrochaites. S. pennellii*, which has green fruits, is distributed along the western coast of Peru and is morphologically quite distinct from other tomatoes. The leaflets are often covered in gray pubescence and have round margins. The species has been described as nearly exclusively outcrossing. Exceptions comprise the southernmost accessions LA716 and LA2963, which are entirely self-compatible (Mutschler and Liedl, 1994), and LA1941, which features 91% self-compatible individuals (Rick and Tanksley, 1981).

Accession LA716 was used as a parent to create a population of 76 ILs, with large chromosomal segments of *S. pennellii* (LA716) introgressed into the M82 *S. lycopersicum* background (Eshed *et al.*, 1992). This IL population has been used to define many QTLs (Lippman *et al.*, 2007) and dissect traits such as metabolic and volatile profiles (Schauer *et al.*, 2006; Goulet *et al.*, 2015), sugar yield and flavor (Fridman *et al.*, 2004; Liu *et al.*, 2016), and whole-plant traits such as photosynthetic capacity (de Oliveira Silva *et al.*, 2018). In the case of sugars, this population was instrumental in identifying *Lin5*, an invertase that yielded higher glucose and fructose levels (Fridman *et al.*, 2000, 2004). Later, it was also found as a causative sugar modifier in a red-fruited tomato population (Tieman *et al.*, 2017).

The value of this population has recently been enhanced by creating several hundred sub-ILs to further break down genomic segments of *S. pennellii* (Alseekh *et al*., 2013), thus increasing the genetic resolution. A BIL population comprising 446 lines carrying an average of 2.7 introgressions was derived from the same parents as the ILs (Ofner *et al.*, 2016), providing an additional valuable genetic resource for *S. pennellii.* Furthermore, an individual of accession LA716 was chosen for sequencing based on its low heterozygosity due to selfing and its importance in genetic studies such as the IL population (Bolger *et al.*, 2014). This also shed light on its drought tolerance, which appears to be associated with transposable element insertions and the cuticle. Early studies involving *S. pennellii and S. lycopersicum* helped to identify the *fw2.2* fruit size QTL (Alpert and Tanksley, 1996; Frary *et al.*, 2000), which was later cloned (Frary *et al.*, 2000).

Metabolomic analysis in *S. pennellii* revealed high levels of the non-proteogenic amino acid γ-aminobutyric acid, two organic acids (citric and malic), and the stress metabolite *myo*-inositol in the fruit, as well as the anti-herbivory metabolite chlorogenic acid (Schauer *et al.*, 2005). It also showed both qualitative and quantitative variation in phenylpropanoid levels (Tohge *et al.*, 2020) and qualitative changes in the levels of SGAs (Schwahn *et al.*, 2014). Some of these traits were partially uncovered following the reanalysis of previous QTL studies of the ILs (Alseekh *et al.*, 2015), but further work is needed to formally identify the genomic basis of these differences. In the same vein, a *S. pennellii* BIL population (Ofner *et al.*, 2016), similar to that described for *S. neorickii* above, may provide more insight because it has only been characterized thus far for select regions (Fan *et al.*, 2016) or for studies of tomato peel (Szymański *et al.*, 2020).

LA5240, the ‘lost accession’ probably representing LA2963 (Schmidt *et al.*, 2017), has been used to generate two BIL populations with two modern inbreds and comprises 500 and 1400 lines in the BC2F6-8 generation (Torgeman *et al.*, 2024). These lines have been used to understand heterosis (Torgeman and Zamir, 2023). Furthermore, because LA5240 shows resistance to tobamovirus tomato brown rugose fruit virus (ToBRFV), this population has been used to map resistance QTLs and has uncovered a QTL close to *tomato mosaic-1* (*Tm-1*) (Rochsar *et al.*, 2025). A long-read genome sequence is available for this accession (Schmidt *et al.*, 2017), so these new backcross populations will be invaluable for the study of many additional traits. This new *S. pennelli* population has the advantage over the LA716 population that it does not carry a necrotic dwarf trait found in LA716 (Torgeman and Zamir, 2023), potentially accelerating trait discovery.

The second species in the *Hirsutum* group (*S. habrochaites*) also has green fruits and probably diverged from its sister species *S. pennellii* about 1.8 Mya (Yu *et al.*, 2022). This species is distributed across northern Peru and southern Ecuador and the plants are quite large and robust, with the largest leaflets in the wild tomato clade (Peralta *et al.*, 2005). It shows a range of reproductive modes, with self-compatible, self-incompatible, and mixed populations (Broz *et al.*, 2017). The populations with lower degrees of outcrossing are located on the periphery of the species range (Rick, 1983). Recently, selfing was shown to have evolved independently in the northern and southern populations (Markova *et al.*, 2016) where different mutations might have led to the loss of self-incompatibility (Broz *et al.*, 2017). The metabolomic studies described above revealed that *S. habrochaites* contains high levels of threonine and tryptophan, as well as citrate and malate (Schauer *et al*., 2005). It also contains high levels of the phenylpropanoid isorhamnetin and flavonol glycosides (Tohge *et al*., 2020) and, like *S. neorickii*, it has some highly abundant SGAs that are typical of the species (Schwahn *et al*., 2014). Furthermore, *S. habrochaites* exhibits a high fructose-to-glucose ratio, which was mapped to a causal sugar transporter *SWEET* gene (Shammai *et al.*, 2018). Some accessions of *S. habrochaites* are resistant to whitefly and contain higher levels of sesquiterpenes (Rutz *et al.*, 2024).

Accession LA1777 of *S. habrochaites* has been used to generate a population of near isogenic lines (NILs) and BILs with domesticated tomato. This population has 57 core lines, as well as additional lines (Monforte and Tanksley, 2000) that have been characterized for fruit volatiles (Mathieu *et al.*, 2009) (Table 2). However, this resource remains under-characterized at the metabolomic level, despite recent genome analysis revealing a potential expansion of terpene synthase genes (Yu *et al.*, 2022).

## More distant relatives

There are two tomato-like species in *Solanum* section *Juglandifolium* (*Solanum ochranthum* and *Solanum juglandifolium*) and two more in *Solanum* section *Lycopersicoides* (*Solanum lycopersicoides* and *Solanum sitiens*), which are recognized as sisters to section *Lycopersicon* (Peralta *et al*., 2008). All four species have yellow flowers like tomato, but they lack sterile anther appendices (Albrecht and Chetelat, 2009). When Rick evaluated the compatibility among *S. sitiens*, *S. lycopersicoides*, *S. ochranthum*, and several tomato species with red and green fruits, he established that *S. lycopersicoides* is compatible with several tomato species, whereas *S. sitiens* is compatible with *S. lycopersicoides* (Rick, 1979). All crosses of *S. ochranthum* to the other tomato species initially failed (Rick, 1979).

Members of section *Juglandifolium* are found in wet climates and they resemble woody perennials, reaching impressive lengths (Rick, 1988). Their fruits are larger than those of any of the wild tomatoes and emit an apple-like fragrance, but they take a very long time to mature (Rick, 1988). A genetic map based on a cross between *S. juglandifolium* and *S. ochranthum* revealed that section *Juglandifolium* shares a chromosomal inversion of 10L with section *Lycopersicon*, but not with section *Lycopersicoides*, which represents the ancestral state (Albrecht and Chetelat, 2009). However, tomato has a reciprocal translocation of chromosomes 8 and 12 compared with one of the *Juglandifolium* species.

Members of section *Lycopersicoides* (*S. lycopersicoides* and *S. sitiens*) grow in arid regions of southern Peru and northern Chile (Chetelat *et al*., 2009) and are best described as shrub or sub-shrub species (Peralta *et al.*, 2008). These species have also been collected from high altitudes (Chetelat *et al.*, 2009). Both species are highly resistant to abiotic stress, but *S. sitiens* is found in drier habitats than *S. lycopersicoides* (Rick, 1988) and tolerates drought and salt stress as well as freezing (Rick, 1988; Chetelat *et al.*, 2009). Indeed, *S. sitiens* has several xerophytic characteristics, including thick leaves and fruits that desiccate during maturation (Rick, 1988; Chetelat *et al.*, 2009). *S. lycopersicoides* also shows cold tolerance, as well as resistance to cucumber mosaic virus and leaf mold (Zhao *et al.*, 2005).

A metabolic analysis of an introgression population (Canady *et al.*, 2006) combined with the genomic analysis of the black-fruited *S. lycopersicoides* parent LA2951 revealed loci for phenolics and carotenoids, including an underlying candidate gene for carotenoids (Powell *et al.*, 2022). Furthermore, syntenic analysis of R2R3-MYB transcription factor genes shed light on the identity of the *Aubergine* locus underlying anthocyanin production in this black-fruited species. In addition, it was used to identify the gene conferring resistance against certain *Pseudomonas* and *Ralstonia* strains (Mazo-Molina *et al.*, 2020) (Table 2).

A recent IL population in which LA4331 (wild *S. sitiens* parent) was used for introgression into the fresh market line LA4354 (Chetelat *et al.*, 2019), supported by the reference genome for LA1974 (Molitor *et al.*, 2021), will help to identify QTLs for stress adaptation and metabolism.

## Future challenges and breaking barriers

Given the genomic breakthroughs outlined above, and the diversity present in the tomato clade, multiple exotic alleles and genes have been identified and are already being used in breeding. However, this has often been driven by the availability of (i) natural accessions that are studied for their behavior, genetic diversity, and individual phenotypes, as well as (ii) the generation of powerful mapping populations ranging from RILs to IL populations exhibiting individual advantages and disadvantages. Particularly for the more exotic germplasm, generating elite varieties can take time, unless gene-editing techniques can be used to transfer beneficial alleles from exotic material to elite lines.

Genomics and bioinformatics have made massive progress, and we can now make use of populations to introgress germplasm even from outside the tomato clade (i.e. *S. lycopersicoides* and *S. sitiens*). However, generating these populations requires extensive crossing efforts, and introgression into breeding material often results in excessive linkage drag, for example, for virus resistance (van Rengs *et al.*, 2022). Furthermore, exotic germplasm still necessitates specifically tailored bioinformatic solutions to account for introgressed portions when analyzing transcriptomic responses (Powell *et al.*, 2022). However, the latter problem is likely to be solved with the widespread adoption of such exotic germplasm across different plant communities.

The difficulties encountered in generating populations can also be overcome, at least in the case of breaking intraspecific reproductive barriers between cultivated tomato and its wild relatives, which hamper introgressing exotic germplasm. Recent advances in mapping approaches have greatly improved our understanding of underlying genes and QTLs (Qin *et al.*, 2018; Jewell *et al.*, 2020; Qin and Chetelat, 2021), which might allow biotechnological adaptations to overcome these barriers.

Recent price reductions and advances in sequencing techniques are now enabling studies of recombination as well. As an early example in tomato, RIL populations were subjected to low-coverage sequencing to identify recombination regions. These studies have revealed that recombination-rich regions are often associated with AT-rich motifs (de Haas *et al.*, 2017). Existing genome sequencing data have then been used to assess historical recombination patterns, showing a largely conserved recombination landscape in different domestic and wild populations (Fuentes *et al.*, 2022).

In the tomato clade, interspecific recombination is typically lower than intraspecific recombination. For the exotic germplasm used, recombination rates are somewhat negatively correlated with the degree of sequence divergence. For example, the recombination rate in *S. lycopersicoides* × *S. lycopersicum* hybrids is approximately 10 times lower than in intraspecific crosses (Canady *et al.*, 2006). Recent technological advances now enable the sequencing of individual pollen cells to directly detect crossover events (Rommel Fuentes *et al.*, 2020; Castellani *et al.*, 2024; Zhang *et al.*, 2025). This approach has been applied to several interspecific crosses between domesticated tomato and wild relatives, leading to the identification of recombination cold spots, that is, regions where crossover events are infrequent (Fuentes *et al.*, 2024). These cold spots can significantly hinder breeding efforts. Whereas some may be attributed to structural variations, others were associated with gypsy and copia transposable elements and, notably, with certain resistance (*R*) genes (Fuentes *et al.*, 2024). Identifying such recombination cold spots is crucial for selecting optimal breeding material that combines beneficial traits while minimizing structural barriers to crossover. Alternatively, targeted genome editing offers the potential to reshape the recombination landscape, as has been demonstrated in Arabidopsis (Schmidt *et al.*, 2020).

However, given the availability of the genomic resources within the tomato clade and the possibility to reconstruct genes that have been lost during domestication and improvement (Gao *et al.*, 2019), together with the availability of CRISPR/Cas9 gene editing and conversion technologies, totally new opportunities arise. As it has already been demonstrated that domestication can be recapitulated (Lemmon *et al.*, 2018) by introducing already-known beneficial traits and thus *de novo* domesticating wild species, a logical next step would be to tailor plants based on specific needs and breeding targets. As these might be different depending on the downstream use (e.g. in the simplest case, tomatoes for processing versus direct consumption), this would require a set of different tomatoes to be generated. First, this would require unlocking larger populations of wild tomatoes, both genomically and phenotypically, to gain a comprehensive overview of traits and adaptation to different ecological niches of wild tomatoes. Then it would require choosing the best germplasm and deciding from which genotypes to start. In the case of potato, a recent approach aimed at developing ideal haplotype sets for potato improvements (Cheng *et al.*, 2025). Here one can envision that AI-based approaches will help speed up breeding efforts and optimize strategies for selecting germplasm considering complex genetic interactions in the future. While AI is already aiding in analyzing genomic data in terms of genes, transposable elements, and their annotation, an AI-based understanding of gene networks is starting to develop. For example, it is possible to predict gene expression based on the genomic context for tomato and other plants (Peleke *et al.*, 2024). The next logical step is the adaptation of expression by tailored genomic changes identified by AI. Here the rapid development of AI-based approaches, together with the massive decrease in costs for genomic and transcriptomic data generation, will be extremely beneficial to further train AI-based tools on different population data first to predict expression behavior and from there to predict more complex phenotypes.

### Making better use of the growing data resources

Since the initial genome release of the Heinz tomato cultivar, genomic (and phenotypic) information has expanded rapidly. The tomato community was greatly aided by the availability of the Tomato Genetics Resource Center resource providing easy and straightforward access to wild tomato relative germplasm, and the Sol Genomics Network (Fernandez-Pozo *et al.*, 2015) providing a community aggregation point and access to public and pre-released genomics data. It is with the advent of ever larger populations that the lessons learned so far, namely, that data need to be well organized, accessible, and reusable, will become ever more important. This will be important not only for the growing tomato community, but especially for training AI to uncover novel associations. To manage these data must necessarily encompass FAIR (findable, accessible, interoperable, and reusable) plant data management (Wilkinson *et al.*, 2016; Weil *et al.*, 2023) to allow AI training, but also to readily select traits and lines for analysis. This will likely necessitate additional and even more integrated data resources that bring genomic, phenotypic, and even ecological data together.

## Conclusion

Recent technological advances have allowed us to determine whether classical taxonomic assignments are supported by corresponding evidence of genetic divergence, and genomic data have shed light on the domestication history of modern tomato in unprecedented detail. Genomic data coupled with wild population collections and genetic resources in the form of ILs and MAGIC populations have allowed us to map multiple quantitative traits, including those related to fruit quality and stress tolerance. The rate of these advances is accelerating, and the availability of donors of exotic germplasm with desirable tolerance traits provides a valuable opportunity to transfer the corresponding genetic components using genome editing approaches such as CRISPR/Cas9 (Wang *et al.*, 2024).

Box 1. **Genome assemblies using latest genomic technologies**

Historically, several genomes were in **draft stage** and often fragmented, where multiple fragments would represent an entire chromosome. However, these fragments can be ordered and connected by gaps (represented by stretches of ‘N’) by using genetic maps or, more recently, optical mapping or molecular techniques to unravel chromatin conformation, such as Hi-C. The latter chromatin conformation techniques allow the detection of chromatin interactions and reveal information on the proximity between fragments. This can be used to validate assemblies and/or to join genomic assembly fragments. The latest assembler tools can profit from such data, and tools to combine fragments with such data exist (Zhou *et al.*, 2023). This allows **chromosome-scale assemblies** where almost all the information of the sequence is ‘anchored’ to the relevant chromosomes in the correct order, which is important for breeding and biotechnological approaches.

State-of-the-art sequencing technologies for assemblies comprise both PacBio and nanopore long-read sequencing. Together, these technologies achieve much more contiguous assemblies, in the ideal case, that stretch from ‘**telomere to telomere**’ without gaps. This is important, as these gap-free genomes promise to contain (almost) all genomic information, including genes and transposons. In the tomato clade, it is possible to obtain such assemblies with long read sequencing alone, depending on heterozygosity and repeat structure (van Rengs *et al.*, 2022; Koren *et al.*, 2024). On the other hand, in the case of heterozygous genomes, it is possible to assemble the two haplotypes separately. For (auto)polyploid plants, such as potato, this is much more difficult and requires additional information such as offspring or gamete sequence data (Zhou *et al.*, 2020; Sun *et al.*, 2022; Serra Mari *et al.*, 2024).

## Data Availability

No data provided.
